# Supplementary material for: Lessons Learned from the COVID-19 Pandemic: Interpreting Vaccination Strategies in a Nationwide Demographic Study
Source: Vaccines (Basel). 2024 May 26;12(6):581. doi: 10.3390/vaccines12060581 (PMC11209574; doi:10.3390/vaccines12060581)
Supplement: Supplementary file 1 [file vaccines-12-00581-s001.zip › vaccines-2991881-supplementary.pdf]

Supplementary Table S1. Proportions of vaccinations according to key parameters in individuals administered with two doses from the same manufacturer or two doses from different manufacturers.

Parameter	Same manufacturer	Different manufacturers
Vaccinated	78 727 497	1 063 079
Male sex – N° of vaccinated (%)	35 112 841 (44.6)	489 329 (46.0)
Age category (% of total vaccinated)		
0-4	0.00	0.00
5-9	0.00	0.00
10-14	0.05	0.02
15-19	1.57	1.03
20-24	5.17	3.54
25-29	6.52	4.51
30-34	7.80	5.50
35-39	7.92	8.79
40-44	8.71	16.16
45-49	9.62	16.83
50-54	10.58	22.94
55-59	10.85	16.41
60-64	10.65	3.27
65-69	8.61	1.75
70-74	6.27	0.84
75-79	4.12	1.21
80-84	2.71	0.94
85-89	1.47	0.60
90+	0.81	0.33

Race (% of total vaccinated)*		
Black	4.36	3.91
Brown	18.89	14.97
Indigenous	0.32	0.07
White	37.78	34.66
Manufacturer (1st shot)		
Sinovac/Butantan	40.63	8.58
AstraZeneca/FioCruz/SII	47.91	87.97
Pfizer	11.46	3.43
Specific groups (% of total vaccinated)		
Healthcare workers	13.57	8.35
Indigenous population	0.36	0.02
Region (% of total vaccinated)		
North	6.38	2.99
Northeast	22.70	11.69
Center-West	7.86	5.28
South	17.05	4.56
Southeast	46.00	75.60

* exact data for “yellow” population not available

Supplementary Table S2. Rates of vaccination according to key parameters and number of doses.

Parameter	One dose (incomplete)	Two doses or Janssen	Three doses up to 15 September 2021
Vaccinated	15 181 376	86 353 647	194 009
Male sex – N° of vaccinated (%)	7 336 854 (48.3)	38 918 259 (45.1)	70 329 (36.3)
Age category (% of total vaccinated)			
0-4	0.00	0.00	0.00
5-9	0.00	0.00	0.00
10-14	0.02	0.05	0.00
15-19	3.56	1.45	0.04
20-24	8.91	4.93	0.13
25-29	8.93	6.25	0.20
30-34	8.12	7.56	0.28
35-39	7.98	8.42	0.45
40-44	10.47	9.48	0.48
45-49	11.45	9.71	0.49
50-54	11.45	10.09	0.51
55-59	9.92	10.10	0.58
60-64	6.46	9.71	1.74
65-69	4.31	7.95	2.89
70-74	2.88	5.83	8.09
75-79	2.04	3.83	7.99

80-84	1.62	2.49	11.49
85-89	0.90	1.36	32.89
90+	0.52	0.83	30.80
Race (% of total vaccinated)*			
Black	4.96	4.34	2.88
Brown	22.03	18.75	9.67
Indigenous	0.40	0.30	0.05
White	29.96	37.47	43.58
Manufacturer of 1st dose (% vaccinated)			
Sinovac/Butantan	36.38	37.37	86.48
AstraZeneca/FioCruz/SII	49.40	44.89	2.72
Pfizer	14.22	10.50	0.19
Janssen-Cilag	-	-	-
Specific groups (% of vaccinated)			
Indigenous population	0.43	0.34	0.02
Healthcare workers	8.63	12.48	6.14
Region of the country (% of total vaccinated)			
North	9.34	7.62	0.69
Northeast	28.84	22.30	10.51
Center-West	5.31	7.91	18.13
Southeast	48.39	46.66	65.20
South	8.10	16.84	5.45

* exact data for “yellow” population not available
